# Recent Advances in Noble Metal Nanocatalysts for Suzuki and Heck Cross-Coupling Reactions

**DOI:** 10.3390/molecules15042124

**Published:** 2010-03-25

**Authors:** Radha Narayanan

**Affiliations:** Department of Chemistry, University of Rhode Island, 51 Lower College Road, Kingston, RI 02881, USA; E–Mail: rnarayanan@chm.uri.edu; Tel.: +1-401–874–2298; Fax: +1-401–874–5072.

**Keywords:** noble metal nanoparticles, nanocatalysis, Suzuki cross-coupling reaction, Heck cross-coupling reaction, homogeneous and heterogeneous catalysis

## Abstract

Since metal nanoparticles have a high surface-to-volume ratio and very active surface atoms, they are very attractive catalysts for a wide variety of organic and inorganic reactions, compared to bulk catalysts. Metal nanoparticles suspended in colloidal solutions and those adsorbed onto bulk supports have been used as catalysts for a wide variety of carbon-carbon bond formation reactions such as the Suzuki and Heck cross-coupling reactions. This review article highlights some of the latest advances in the application of noble metal nanoparticles as catalysts for these two industrially important classes of cross-coupling reactions. We will discuss several important advances in using metal nanocatalysts in Suzuki and Heck cross-coupling reactions such as investigations on the nanoparticle shape dependence on the catalytic activity, novel types of supported metal nanoparticles as nanocatalysts, and the use of bi-metallic, tri-metallic and multi-metallic nanoparticles as catalysts for the Suzuki and Heck cross-coupling reactions.

## Introduction to the Suzuki and Heck Cross-Coupling Reactions

**Scheme 1:**
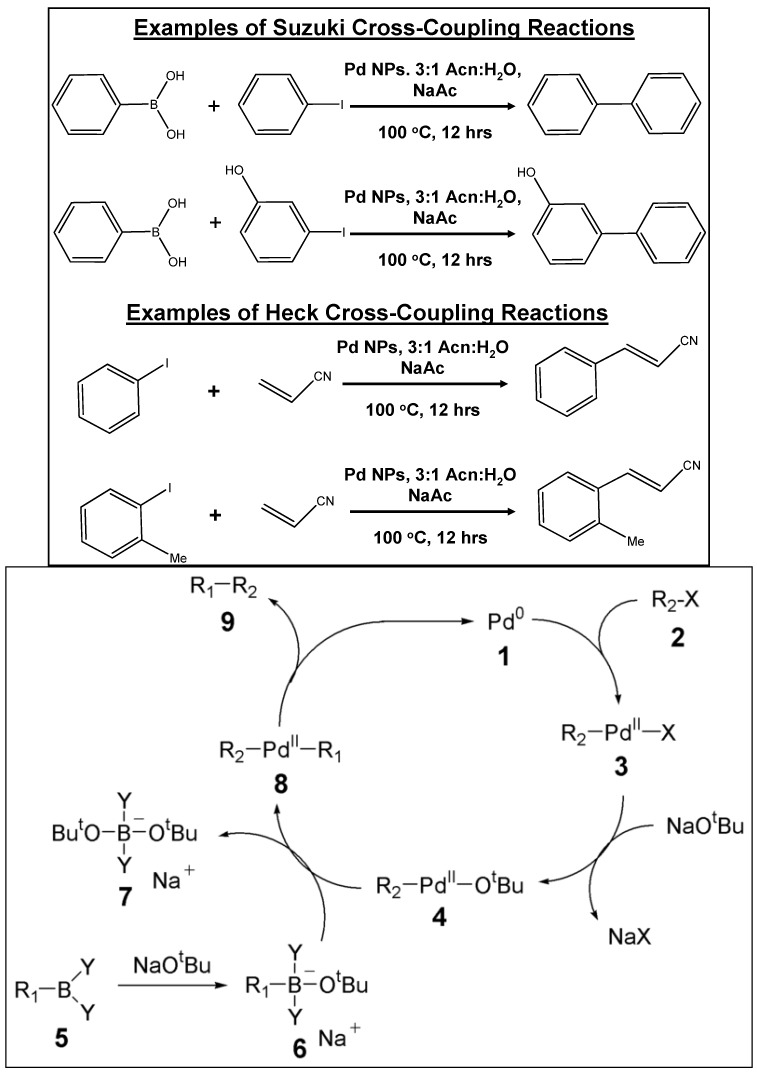
Examples of some types of Suzuki and Heck cross-coupling reactions and the mechanism of the Suzuki reaction.

Suzuki cross-coupling reactions, which are also referred to as Suzuki-Miyaura reactions [1,2,3], involve the cross-coupling of organoboronic acids with aryl halides to form biaryls [3]. The original article that reports the cross-coupling of organoboranes was published in 1979 by Suzuki and Miyaura and hence this class of reactions is often referred to as the Suzuki-Miyaura reaction [4]. In his original paper [4], Suzuki reported on the cross-coupling of alkenyl boronates and alkenyl bromides. Over time, the Suzuki cross-coupling reaction has been further developed to include reactions between boronic acids, boronate esters or organoboranes and organic halides or pseudohalides [5]. Scheme 1 shows a few examples of some basic types of Suzuki cross-coupling reactions. 

**Figure 1:**
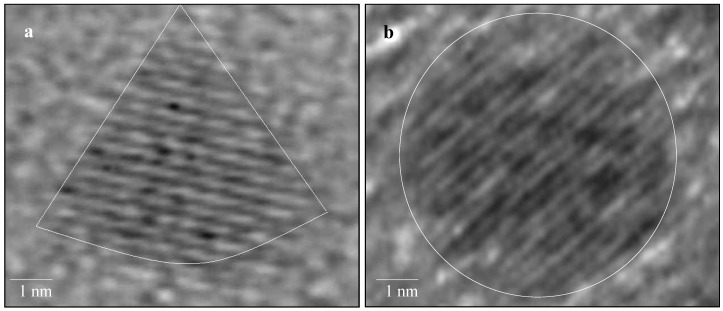
HRTEM image of a tetrahedral nanoparticle (a) and the transformed spherical platinum nanoparticle (b). Reprinted (“Adapted” or “in part”) from [39] with permission. © 2005 American Chemical Society.

Heck cross-coupling reactions [6,7,8] involve coupling of an unsaturated halide with an alkene and are also referred to as Mizoroki-Heck reactions [9]. Heck reactions are very important in industry since substitution reactions can be done on planar centers [10]. The Heck reaction is named after the American chemist, Richard F. Heck who discovered this reaction [10] and T. Mizoroki who also contributed substantially to this field of organic chemistry [11]. Figure 1 also shows a couple of examples of some basic types of Heck reactions. 

### Noble Metal Nanoparticle Catalysts

Metal nanoparticles are very attractive catalysts compared to bulk catalysts since they have a high surface to volume ratios and their surface atoms are very active. Numerous review articles have been written on the use for many different types of organic and inorganic reactions of noble metal nanoparticles suspended in colloidal solutions as well as those adsorbed onto different supports as catalysts. Some types of traditional nanocatalysts include noble metal nanoparticles in colloidal solutions [12,13,14,15,16,17,18,19], those adsorbed onto bulk supports [20,21,22,23,24,25], and lithographically fabricated arrays of nanocatalysts [26,27,28,29,30]. There are numerous articles related to using noble metal nanoparticles as catalysts for the Suzuki and Heck cross-coupling reactions. In this review article, we focus specifically on the recent advances that have been made in the following three areas: nanoparticle shape dependence on the catalytic activity, novel types of supported metal nanoparticles as nanocatalysts, and the use of bimetallic and multi-metallic nanoparticles as catalysts. Some of the recent advances in these areas that have been applied for the Suzuki and Heck cross-coupling reactions will also be discussed in this review.

## Shape Dependence Nanocatalysis for Suzuki and Heck Cross-Coupling Reactions

Nanocatalysis with different metal nanoparticle shapes has become a promising field with the discovery of a facile synthesis of platinum nanoparticles of different shapes via the hydrogen reduction method. In 1996, it was discovered [31] that cubic platinum nanoparticles composed entirely of [100] facets as well as tetrahedral platinum nanoparticles composed entirely of [111] facets can be easily synthesized by using the hydrogen reduction method and varying the ratio of the potassium tetrachloroplatinate precursor platinum salt and the sodium polyacrylate stabilizer. By using a 1:1 ratio of the platinum precursor salt and polyacrylate, cubic platinum nanoparticles were formed and in the case of the 1:5 ratio of the platinum precursor salt and polyacrylate, tetrahedral platinum nanoparticles were formed [31,32,33].

Tetrahedral, cubic, and spherical platinum nanoparticles have been used as catalysts for electron transfer reactions [34,35,36]. The activation energies were obtained for the three different shapes of platinum nanoparticles by monitoring the kinetics during the first 40 minutes of the reaction at different temperatures between 25–40 °C. From the slope of the Arrhenius plot, the activation energies can be calculated for each sample. It was determined that the tetrahedral Pt nanoparticles are the most catalytically active with the lowest activation energy of 14.0 ± 0.6 kJ/mol [34]. The cubic Pt nanoparticles are the least catalytically active with the highest activation energy of 26.4 ± 1.3 kJ/mol. The spherical Pt nanoparticles were treated as near-spherical nanoparticles and were found to have an intermediate catalytic activity and intermediate activation energy of 22.6 ± 1.2 kJ/mol [34].

The tetrahedral platinum nanoparticles were tested as catalysts for the Suzuki cross-coupling reaction between phenylboronic acid and iodobenzene. Recently, it has been reported that platinum complexes such as K_2_[PtCl_4_] can also be used as catalysts for the Suzuki reaction [37]. There has been one study on the use of *spherical* platinum nanoparticles stabilized with TOAF in which it was found that the Pt nanoparticles do not catalyze the reaction [38]. We wanted to test whether the tetrahedral platinum nanoparticles could catalyze the Suzuki cross-coupling reaction between phenylboronic acid and iodobenzene since we have already shown that tetrahedral nanoparticles are more catalytically active than spherical platinum nanoparticles when used as catalysts for the electron transfer reaction between hexacyanoferrate (III) ions and thiosulfate ions [34,39]. It was observed that the tetrahedral platinum nanoparticles can catalyze the Suzuki reaction, but undergoes a transformation in the shape from tetrahedral to spherical shape due to the high temperature that the reaction takes place. Figure 1 shows the HRTEM images of a tetrahedral platinum nanoparticle and a transformed spherical platinum nanoparticle and the lattice fringes of both the tetrahedral and spherical nanoparticle can be seen easily [39]. When the transformed tetrahedral nanoparticles are used to catalyze the second cycle of the Suzuki reaction, it was observed that there is a drastic reduction in the catalytic activity. After the second cycle, the transformed spherical nanoparticles become larger in size due to the Ostwald ripening process [39]. Also, the drastic reduction in the catalytic activity is consistent with previous findings [38] that spherical platinum nanoparticles do not catalyze the Suzuki reaction.

**Figure 2:**
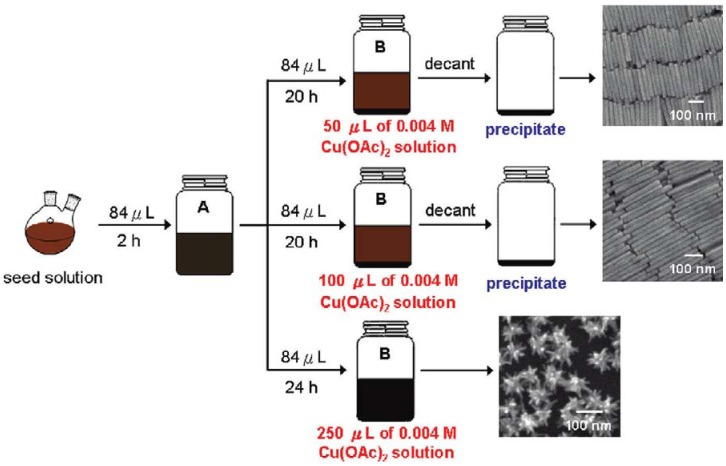
Schematic of the seed-mediated growth process for synthesis of palladium nanorods and branched palladium nanostructures Reprinted (“adapted” or “in part”) with permission from [40]. © 2009 American Chemical Society.

The above studies have opened up many possibilities of synthesizing noble metal nanoparticles of different shapes and testing their catalytic activity for different types of cross-coupling reactions. There have been some important studies in which noble metal nanoparticles of different shapes have been used as catalysts for Suzuki cross-coupling reactions. It has been shown that palladium nanorods and branched palladium nanostructures are good recyclable catalysts for Suzuki cross-coupling reactions [40]. The palladium nanorods were synthesized by using a two-stage seeding growth process in which CTAB capped spherical palladium nanoparticles are prepared by using the sodium borohydride reduction method to reduce the PdCl_2_ into the zero-valent form. The formation of the palladium nanorods involves a two-stage seeding growth process as shown in Figure 2 in which the growth solution consists of CTAB more palladium precursor salt and ascorbic acid which is a mild reducing agent [40]. The spherical palladium nanoparticle seeds were added to the growth solution and this is followed by addition of copper acetate and more ascorbic acid. By modifying this synthetic procedure, different lengths and aspect ratios of the palladium nanorods as well as the branched palladium nanostructures can be achieved [40]. Palladium nanorods with an average length of ~200 and ~300 nm as well as diameters of ~20 have been synthesized by using this method. As a result, the palladium nanorods have an aspect ratio of 10–15 or greater. 

The palladium nanorods and branched palladium nanostructures were tested as catalysts for the Suzuki cross-coupling reaction between phenylboronic acid and iodobenzene [40]. It was observed that the palladium nanorods and branched palladium nanostructures are highly efficient and recyclable catalysts. The palladium nanorods and branched palladium nanostructures were tested as catalysts for the initial Suzuki reaction between phenylboronic acid and iodobenzene to form biphenyl, 2^nd^ cycle of the Suzuki reaction, and also the 3^rd^ cycle of the reaction. Biphenyl yields of 90–92% were obtained for the initial Suzuki reactions and good yields of 82–85% were obtained after the 3^rd^ cycle of the Suzuki reaction. Both of these nanostructures maintain their morphology after the first and second cycle of the reaction and still maintain good biphenyl product yields [40].

**Figure 3:**
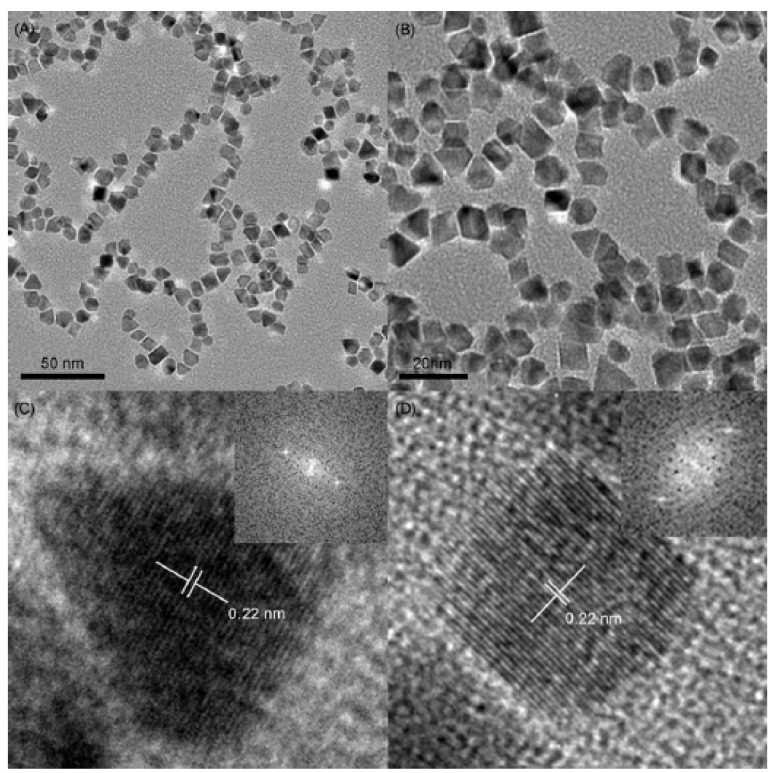
TEM images of triangular and rectangular shaped palladium nanoparticles Reprinted from [41] with permission from Wiley-VCH Verlag GmbH and Co., © 2009.

Palladium nanoparticles of different shapes have also been synthesized in the presence of the precursor palladium salt solution and tri-block Pluronic copolymer solution and stirring vigorously [41]. The size and shape of the palladium nanoparticles can be controlled by varying the pH of the solution resulting in the formation of dendritic, rectangular, and triangular shaped palladium nanostructures [41] as can be seen in Figure 3. The catalytic activity was determined for these different shaped palladium nanoparticles as catalysts for the Suzuki cross-coupling of 4-bromoacetophenone and phenylboronic acid [41]. Using these palladium nanoparticles as catalysts resulted in high catalytic activity and very high yields (91–99% yield) of the desired products.

## Novel Types of Supported Metal Nanocatalysts for Suzuki and Heck Cross-Coupling Reactions

There have been many different types of support materials that have been used for adsorption of noble metal nanoparticles such as biological supports [42], magnetic nanostructures [43], carbon nanotubes [44,45], and polymers [41]. The noble metal nanoparticles adsorbed on many of these types of supports have been used as catalysts for both the Suzuki and Heck cross-coupling reactions. 

Bio-generated palladium nanoparticles that are formed on the surface of Gram-negative bacteria have been used as catalysts in both Suzuki and Heck cross-coupling reactions [42]. In this type of synthesis, the palladium precursor source was added to the cells suspended in anaerobic MOPS buffer after growth and harvesting of the Gram-negative bacteria. Then, formate, an electron donor, is added and the reduction takes place within a few hours in which the color changed from light orange to black. The particle size distribution of the palladium nanoparticles generated using this method is very polydisperse. In the case of the palladium nanoparticles supported on Gram-negative bacteria, very high product yields between 85–100% product yields were obtained for a wide variety of Suzuki and Heck cross-coupling reactions [42]. The authors believe that this type of unique biological support for loading noble metal nanoparticles can be easily extended to many other carbon-carbon bond formation reactions in organic chemistry since it is a practical and simple means of sustainable organometallic chemistry. This method also represents a green alternative compared to conventional methods of recovering important noble metals from wastes [42].

**Figure 4:**
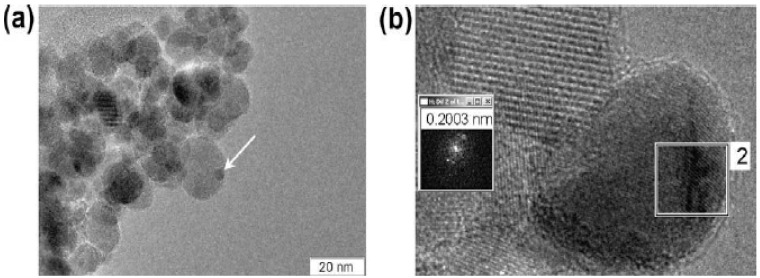
TEM image showing the palladium nanoparticles adsorbed onto the magnetic nanoparticles (a) and HRTEM image showing the lattice fringes of the palladium nanoparticles as well as the electron diffraction pattern observed (b) Reprinted from [43] with permission from Elsevier, © 2009.

Magnetic nanoparticles have also been used as support materials for adsorption of palladium nanoparticles [43]. The palladium nanoparticles loaded onto the magnetic nanoparticle supports have been used as catalysts for the Suzuki reaction between phenylboronic acid and bromobenzene as well as the Heck reaction between styrene and 4-bromonitrobenzene. Based on the kinetics of the reactions, it was determined that the reaction is not diffusion limited, but is influenced by the chemical nature of the aryl bromide. These supported nanoparticles have consistently resulted in very high yields of products (85–100% yield) of a variety of Suzuki and Heck cross-coupling reactions as well as other types of reactions such as hydrogenations. Figure 4 shows a TEM image showing where the palladium nanoparticles are adsorbed onto the magnetic nanoparticles as well as a HRTEM image showing the lattice fringes of the palladium nanoparticles adsorbed onto the magnetic nanoparticles [43]. It was found that the catalysts retained their catalytic activity for several cycles, but the reaction rate is lower upon recycling.

Spherical palladium nanoparticles have been immobilized onto nanometer-scale platelets of montmorillonite (MMT) [46] via cation exchange of Pd^+2^ for Na^+^ followed by chemical reduction [47]. The size of the Pd-MMT nanocatalysts is 6.4 ± 1.2 nm. The palladium nanoparticles supported on organoclays are easily dispersed in water as well as polar solvents. This resulted in new types of catalysts with easy polarity design. These Pd-MMT nanocatalysts have been tested for various Suzuki cross-coupling reactions as well as hydrogenation reactions. When the Pd-MMT nanocatalysts are used as catalysts in several Suzuki cross-coupling reactions, the product yields are between 86–98%. It is also worth noting that it is not easy to recycle these catalysts and this is believed to be due to Pd surface poisoning.

**Figure 5:**
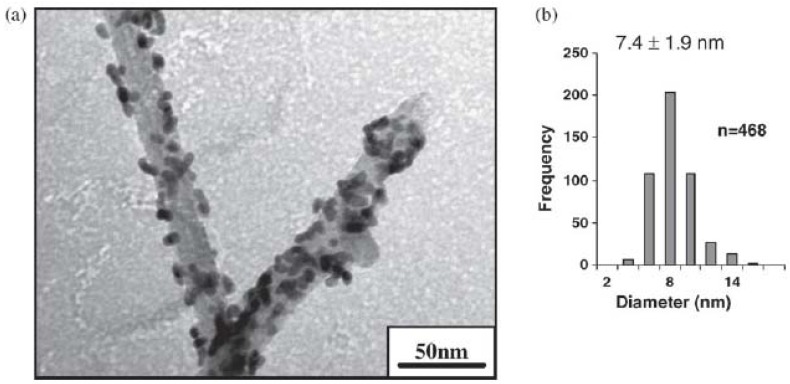
TEM image of the Pd-DMAP nanoparticles bound to MWCNTs (a) Size distribution of the palladium nanoparticles bond to the MWCNTs (b). Reprinted from [44] with permission from Elsevier, © 2009.

Noble metal nanoparticles have also been adsorbed onto carbon nanotubes in which the carbon nanotubes serve as the support materials [44.45]. The palladium nanoparticles can be immobilized onto functionalized multi-wall carbon nanotubes (MWCNTs) through displacement of DMAP, which is the stabilizing ligand. The process by which the immobilization takes place involves the DMAP being displaced by thiolated molecules chemically attached to the MWCNTs. The metal-thiol bond is a very strong bond and in this case, the Pd-S bond is formed when the palladium nanoparticles are covalently attached to the MWCNTs. Figure 5 shows the TEM images of the Pd-DMAP nanoparticles bound to MWCNTs [44]. In addition, the size distributions of the palladium nanoparticles bound to the MWCNTs is also shown in Figure 5 and it can be seen that the size of Pd-DMAP nanoparticles is 7.4 ± 1.9 nm. The resulting nanocomposite, Pd-MWCNTs, is an active and recyclable catalyst maintaining high catalytic activity even in the 5^th^ cycle of the reaction. These nanocomposites have been tested for many different Suzuki reactions such as the coupling of three different types of 4-halobenzoic acids and phenylboronic acid and it was found that older dispersions of the nanocomposites are more active catalysts than freshly prepared dispersions of the nanocomposites [44]. Since the palladium nanoparticles are bound to the MWCNTs covalently, the Pd-MWCNTs nanocomposites serve as recyclable composite catalysts.

Recently, the Suzuki reaction and other palladium catalyzed reactions have become very important and a fixed bed reactor loaded with Pd nanoparticles is very suitable for C-C bond formation reactions. Monolithic polyionic polymers have been used as supports for the immobilization of noble metal nanoparticles [48,49]. The palladium nanoparticles have been loaded onto the monolithic polyionic polymers and used in continuous flow conditions for a variety of C-C bond formation reactions such as the Suzuki and Heck cross-coupling reactions. The coupling of 4′-bromoacetophenone and phenylboronic acid was used as a model reaction for optimizing the process. After optimizing the reaction conditions such as flow rate, temperature, solvents, *etc*., several other cross-coupling reactions were conducted successfully with reasonably good product yields (60–99% yield) after short reaction times [48].

Perfluoro-tagged palladium nanoparticles supported on fluorous silica gel have been used as catalysts for the Suzuki cross-coupling reactions [50]. It is a surprising finding that fluorinated compounds can stabilize nanoparticles. It is known that perfluorocarbons have low polarizability and as a consequence of this, they have very weak intermolecular dispersion interactions and a very low surface tension since they have very small attractive interactions among themselves and other materials [50]. Nevertheless, it has been reported that fluorinated compounds can indeed stabilize nanoparticles [51,52]. The perfluoro-tagged palladium nanoparticles have an average size of 2–3 nm. The perfluoro-tagged palladium nanoparticles were adsorbed onto fluorous silica gel and used as catalysts for the Suzuki cross-coupling reaction. Moderate to high yields of biphenyl (12–99% yields) are formed in many different conditions such as the solvent, type of base, temperature, and surfactant additives. It is observed that the Suzuki reaction can be conducted in water as solvent and still obtain very high yields [50]. Using water as the solvent to conduct the Suzuki reaction is very environmentally friendly and is an important component of green chemistry.

## Advances in Bimetallic and Multi-Metallic Nanocatalysts for Suzuki and Heck Cross-Coupling Reactions

**Figure 6:**
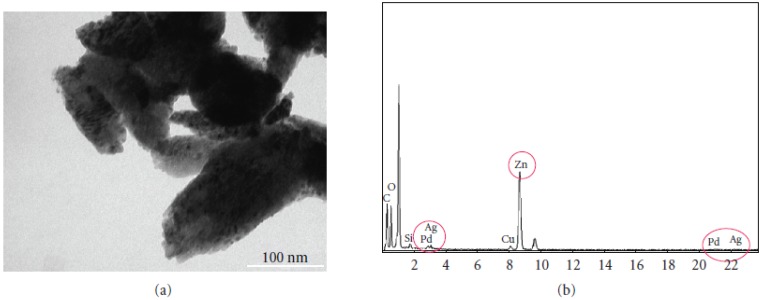
TEM image and EDS spectra for the Pd-Ag bimetallic nanoparticles supported on ZnO Reprinted from [53] with permission from Hindawi Publishing Corporation, © 2009.

There have been several reports on the use of bimetallic, tri-metallic, and multi-metallic noble metal nanoparticles as catalysts for different types of cross-coupling reactions such as the Suzuki and Heck cross-coupling reactions [53]. Different types of Pd based bimetallic nanoparticles loaded on ZnO nanopowder have been synthesized and tested as catalysts for the Suzuki cross-coupling reactions and hydrogenation reactions. The specific types of bimetallic nanoparticles are Pd-Ag, Pd-Cu, and Pd-Ni bimetallic nanoparticles loaded onto zinc oxide nanopowder. The bimetallic nanoparticles were prepared by γ-irradiation at room temperature without using any reducing agents [53]. These catalysts can be easily reused by filtering the final reaction mixture to separate the catalyst from the reaction mixture. Figure 6 shows an example of a TEM image of the Pd-Ag bimetallic nanoparticles loaded on ZnO nanopowder [53]. 

The EDS spectra were obtained for the Pd-Ag bimetallic nanoparticles loaded on ZnO nanopowder and it can be seen that the Pd, Ag and Zn peaks are present indicating that both palladium and silver are present in the sample. Moreover, the presence of the Zn peaks confirms that the bimetallic nanoparticles are loaded onto the ZnO nanopowder. The authors have successfully synthesized the Pd-Cu and Pd-Ni bimetallic nanoparticles and characterized these samples in a similar manner with TEM and EDS. It was found that the catalytic efficiency decreases in the order of Pd-Ag/ZnO > Pd-Cu/ZnO > Pd-Ni/ZnO > Pd/ZnO. The Pd-Ag/ZnO catalyst has product yield of 98.9% during the first cycle of the Suzuki reaction and 81.5% after the fifth cycle of the reaction which shows that they are good catalysts for the Suzuki reaction. Also, these catalysts can be applied for other cross-coupling reactions such as Heck and Stille reactions [53].

The bimetallic nanoparticles supported on carbon have also been used as catalysts for the Suzuki and Heck cross-coupling reactions [54]. The Pd-Ag, Pd-Ni, and Pd-Cu bimetallic nanoparticles were also synthesized by γ-irradiation method at room temperature without the use of any reducing agents. After the bimetallic nanoparticles are synthesized, they are then loaded onto the carbon support. These catalysts as well as Pd/C, which are palladium nanoparticles loaded onto the carbon supports, are used for a variety of Suzuki and Heck cross-coupling reactions. In the Suzuki and Heck cross-coupling reactions, the catalytic efficiency based on the yield of the desired product decreases in the following order: Pd-Cu/C > Pd/C > Pd-Ag/C > Pd-Ni/C [54]. In the case of the Pd-Cu/C bimetallic nanoparticles, there is 97.7% biphenyl yield, and after the fifth recycle, the biphenyl yield is 90.5% which shows that these catalysts have good recycling potential.

**Figure 7:**
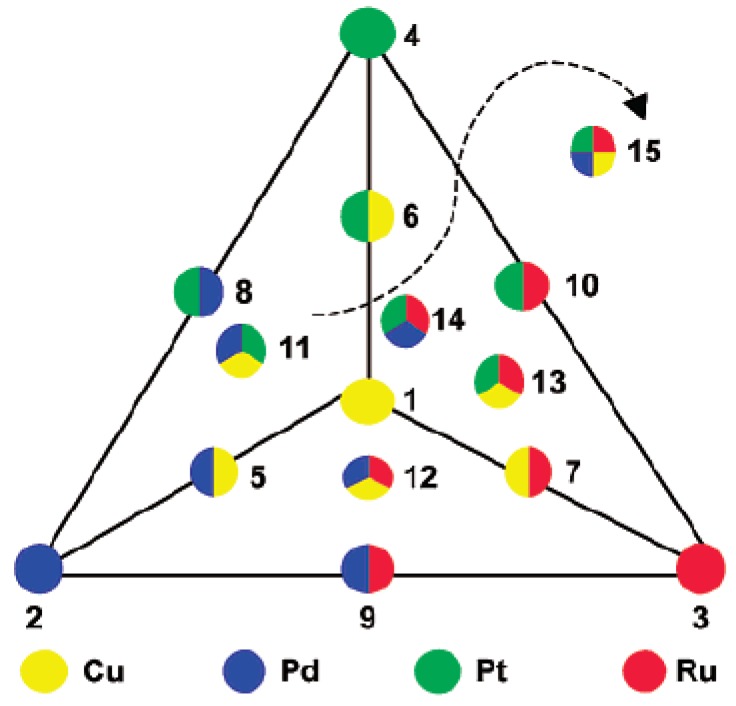
Experimental schematic of the different combinations of monometallic, bimetallic, tri-metallic and multi-metallic nanoparticles synthesized. Reprinted (“adapted” or “in part”) with permission from [38], © 2002 American Chemical Society.

A wide variety of bimetallic, tri-metallic, and tetra-metallic nanoparticles have been used as catalysts for Suzuki cross-coupling reactions [38,55]. Copper based alternative nanocatalysts are tested as catalysts for the Suzuki cross-coupling reactions. The different types of nanoparticles were prepared by mixing predetermined quantities of the appropriate homogeneous stock solutions of the metal chloride precursors, followed by reduction with tetraoctylammonium formate (TOAF) in dimethylformamide (DMF). Figure 7 illustrates the many different combinations of bi-metallic, tri-metallic, and tetra-metallic nanoparticles that are tested as catalysts for the Suzuki cross-coupling reaction [38]. Kinetic studies of the biphenyl yields as a function of the reaction time were plotted for the different combinations of noble metal nanoparticles (monometallic, bimetallic, tri-metallic, and multi-metallic) as nanocatalysts for the Suzuki cross-coupling reactions. 

In the case of the model Suzuki reaction involving cross-coupling of phenylboronic acid and iodobenzene, the use of palladium nanoparticles resulted in the highest catalytic activity among the monometallic nanocatalysts. It was observed that there is no reaction when platinum nanoparticles are used as the catalyst, but ruthenium and copper nanoparticles are active and stable. Among the bimetallic nanocatalysts, the Pd-Cu nanoparticles had the highest catalytic activity being close to that of the Pd nanoparticles by themselves. This shows that the presence of copper also results in high catalytic activity for the Suzuki reaction between phenylboronic acid and iodobenzene. Among the tri-metallic nanocatalysts, the highest catalytic activity was exhibited by Cu-Pd-Ru tri-metallic nanoparticles. This also shows that the palladium and ruthenium are active and stable when used as tri-metallic nanocatalysts for the Suzuki cross-coupling reaction. The tetra-metallic nanocatalyst, Cu-Pd-Pt-Ru, was less active than the three types of tri-metallic nanoparticles, but was stable and reached 100% conversion to the biphenyl product after 24 hours. The lower activity for the tetra-metallic nanocatalyst is attributed to structural differences due to the presence of platinum. Overall, it has been shown that the copper based colloids could be an inexpensive and environmentally friendly alternative to that of more expensive noble metal nanocatalysts.

## Summary and Future Outlook

Noble metal nanoparticles have a high surface-to-volume ratio which makes their surface atoms very active, compared to bulk catalysts. The Suzuki and Heck cross-coupling reactions are both very industrially important carbon-carbon bond formation reactions that are catalyzed with noble metal precursor salts as well as noble metal nanoparticles. This review article highlights many important advances in the area of shape-dependent nanocatalysis, the use of novel types of supports for loading the noble metal nanoparticles, and the use of bi-metallic, tri-metallic, and tetra-metallic nanoparticles as catalysts for the Suzuki and Heck cross-coupling reactions. All three areas are very important areas in nanocatalysis and the future looks bright and ripe for further discoveries. In addition, there are also many other important recent advances that haven taken place in the field of nanocatalysts for Suzuki and Heck cross-coupling reactions such as the use of gold nanoparticles and bimetallic indium nanostructures as catalysts for the Suzuki reaction [56,57,58,59,60,61,62].

Shape dependent nanocatalysis is a relatively new field from the 1990s onward and there has been great interest in applying the use of different shaped palladium nanoparticles as catalysts for a variety of Suzuki and Heck cross-coupling reactions. The field of supported metal nanoparticles continues to grow at a rapid rate and many interesting types of supports have been used to load or immobilize noble metal nanoparticles. These supported nanocatalysts show high product yields and recyclability among Suzuki and Heck cross-coupling reactions. Last, but not least, the use of bi-metallic, tri-metallic, and multi-metallic nanoparticles is a new area of interest and provides avenues which can result in high catalytic activity while also reducing the cost of the preparation of the catalysts. There will definitely be many more advances in the future and this will result in optimizing the system to achieve the highest catalytic activity while also lowering the production costs of the nanocatalysts.
